# Dietary Patterns are Associated with *Helicobacter Pylori* Infection in Chinese Adults: A Cross-Sectional Study

**DOI:** 10.1038/srep32334

**Published:** 2016-08-30

**Authors:** Yang Xia, Ge Meng, Qing Zhang, Li Liu, Hongmei Wu, Hongbin Shi, Xue Bao, Qian Su, Yeqing Gu, Liyun Fang, Fei Yu, Huijun Yang, Bin Yu, Shaomei Sun, Xing Wang, Ming Zhou, Qiyu Jia, Honglin Zhao, Kun Song, Kaijun Niu

**Affiliations:** 1 Nutritional Epidemiology Institute and School of Public Health, Tianjin Medical University, Tianjin, China; 2 Health Management Centre, Tianjin Medical University General Hospital, Tianjin, China; 3 Collaborative Innovation Center of Non-communicable Disease, Tianjin Medical University, Tianjin, China

## Abstract

Previous studies indicated that food consumption was associated with *Helicobacter pylori* infection, but no study has yet investigated the association between *Helicobacter pylori* infection and dietary patterns. The aim of this study was to evaluate the associations between *Helicobacter pylori* infection and dietary patterns in Tianjin, China. The final cross-sectional study population comprised 10407 participants. Dietary consumption of participants was assessed via food frequency questionnaire. Factor analysis was used to identify dietary patterns, and *Helicobacter pylori* infection status was diagnosis by *H. pylori* urease Immunogold Testing kit. Participants in the highest quartile of the high-carbohydrate/sweet pattern showed a multivariable-adjusted OR (95% CI) of 1.65 (1.27–2.17) for the prevalence of *H. pylori* infection compared with those in the lowest quartile. The multiple adjusted OR for scores of the extreme quartile of high-protein/cholesterol pattern was 0.75 (95% CI, 0.57–0.98). This study demonstrated that a diet rich in carbohydrates and sweets was positively associated with the prevalence of *H. pylori* infection; interestingly, a diet characterized by high intake of animal offal, animal blood, fish, seafood, and poultry was associated with a reduction of prevalence of *H. pylori* infection.

*Helicobacter pylori* (*H. pylori*) has been the subject of increased attention in the last 30 years^1^ and affects approximately 50–70% of the population worldwide^2^. The prevalence of *H. pylori* infection ranges from 41.5% to 72.3% and may vary with the population studied and with the geographic area in China^3^. *H. pylori* infection is an important cause of chronic active gastritis and is strongly associated with peptic ulcer disease and gastric cancer^4^ and classified as a Group 1 carcinogen by the World Health Organization (WHO) and International Agency for Research on Cancer (IARC)^5^. Moreover, *H. pylori* infection may be associated with an increased risk for pancreatic disease^6^, lymphoma^7^, and atherosclerosis^8^.

Previous studies examined the effects of different dietary components on risk of *H. pylori* infection. Some foods and components possess anti-*H. pylori* activity, such as bovine milk^9^, cranberry^10^, broccoli sprout^11^, fast food^12^, and virgin oil^13^. Most recently, a review demonstrated that a plenty of dietary components, including specific foods, food components, and food products, were associated with *H. pylori* infection^14^. A case-control study from Malaysia found that frequent consumption of tea (OR: 0.023, 95% CI: 0.01–0.07), frequent use of ‘budu’ or local anchovy sauce (OR: 0.09, 95% CI: 0.1–0.7), and frequent use of local foods ‘pegaga’ or *centenella asiatica* (OR: 0.25, 95% CI: 0.1–0.65) were inversely associated with *H. pylori* infection^15^. However, regular diets consist of complex combinations of foods and nutrients ingested together may act independently or may interact with one another. Therefore, examination of dietary patterns, which assess the effects of overall diet and determined by factor analysis can be proxy indicators of real food consumption and availability, providing a more realistic representation of everyday eating habits^16^. Previous studies demonstrated that dietary patterns were associated with prevalence of gastric cancer^17^,^18^. But no study has yet investigated the association between *Helicobacter pylori* infection and dietary patterns. Previous studies focused on the effect of single food item on *H. pylori* infection instead of the whole diets^9^,^10^,^11^. However, we cannot know how the regular diets in relation to the prevalence of *H. pylori* infections, even though we know the effects of all single food items on *H. pylori* infections because of the interactions between food items and nutrients. In contrast, studies on dietary patterns are meaningful in the area of health education, which means we could give dietary recommendations to participants based on overall dietary patterns instead of single dietary components for prevention and cure. To the best of our knowledge, no study has examined the association between dietary pattern and *H. pylori* infection. We thus designed a cross-sectional study to investigate associations between dietary pattern and *H. pylori* infection in an adult population.

## Results

### Characteristics of participants

A total of 10407 participants in Tianjin were included in this analysis. Characteristics of participants according to *H. pylori* infection status are shown in Table 1. Participants with *H. pylori* infection tended to be men (*P* < 0.0001), older (*P* < 0.0001), have higher BMI (*P* < 0.0001), higher physical activity (*P* = 0.01), higher salt intake (*P* < 0.0001), higher prevalence of metabolic syndrome (*P* < 0.001), and higher prevalence of type 2 diabetes (*P* = 0.03). Participants with *H. pylori* infection also trended to drink alcohol everyday (*P* < 0.01) and have lower education level (*P* < 0.0001).

### Dietary patterns

After varimax rotation, factor analysis revealed three dietary patterns and the main factor loadings of each pattern (Table 2). The three dietary patterns accounted for 28.5% of the variance in total food intake. According to the contribution to the total variation, the three dietary patterns were: factor 1 was defined as high-carbohydrate/sweet pattern and characterized by high intake of candied fruits, cakes, and sugared beverages; factor 2, the balanced pattern, was typified by a balance intake of fruits, vegetables, and soya bean products; factor 3, identified as a high-protein/cholesterol pattern and included high intakes of animal offal, animal blood, fish, seafood, and poultry.

### Dietary patterns and *H. pylori* infection

Associations between dietary patterns and the *H. pylori* infection status are shown in Table 3. The high-carbohydrate/sweet pattern was positively associated with the prevalence of *H. pylori* infection (*P* for trend < 0.001) after adjustment. Participants in the highest quartile of the high-carbohydrate/sweet pattern showed a multivariable-adjusted OR (95% CI) of 1.65 (1.27–2.17) for the prevalence of *H. pylori* infection compared with those in the lowest quartile. The high-protein/cholesterol pattern was associated with a lower prevalence of *H. pylori* infection (*P* for trend = 0.04) after adjustment. However, the association between high-protein/cholesterol pattern and *H. pylori* infection was not strong. The multiple adjusted OR for scores of the extreme quartile of high-protein/cholesterol pattern was 0.75 (95% CI, 0.57–0.98). However, the associations were not significant when adjusting for age, sex, and BMI, but only reached significance upon adjusting for a multitude of other, potential confounders. Thus, the results could be affected by these potential confounders. No significant association was observed between balanced pattern and *H. pylori* infection after adjustment.

The relations between major food group consumption and *H. pylori* infection are presented in Table 4. No significant association was observed between any food groups and *H. pylori* infection after adjustment.

## Discussion

This is the first study to assess the associations between dietary patterns and *H. pylori* infection. Our results demonstrated that the high-carbohydrate/sweet pattern which characterized by high intake of candied fruits, cakes, and ice cream was positively associated with the prevalence of *H. pylori* infection.

Previous studies have reported the positive relationship between carbohydrates with *H. pylori* infection^12^. The mechanism that links carbohydrates to *H. pylori* infection is not clear. It has been shown that carbohydrates intake^19^ was associated with metabolic syndrome. Previous studies showed a low-carbohydrates diet was associated with a decreased risk of type 2 diabetes in Japanese women^20^. A large population study in Japan indicated that *H. pylori* infection was significantly associated with the presence of metabolic syndrome^21^. A case-control study shown that prevalence of *H. pylori* was significantly higher in those diagnosed with diabetes as compared to controls^22^. Accordingly, these results suggested that carbohydrate was linked to *H. pylori* infection. Moreover, we found that most food items of the high-carbohydrate/sweet pattern were raw food products, such as ice cream and fruits. A previous study demonstrated that some types of raw ready-to-eat food, such as fruit salad, maybe the sources of resistant and virulent strains of *H. pylori*^23^. The results suggested that raw food products may serve as one route of transmission of *H. pylori*, meaning that further research is needed to clarify the associations between cooking methods and *H. pylori* infection. The high-carbohydrate/sweet pattern in our study was also associated with high intake of alcohol. Our results agreed with previous studies, suggesting that heavy alcohol consumption is predictively associated with *H. pylori* infection^24^,^25^. One study proposed a hypothesis that acute and chronic alcohol consumption disrupts the gastric mucosal barrier and increases the mucosa’s permeability, resulting in chemical inflammation. Subsequently, macrophages and neutrophils release the cytokine interleukin-8 (IL-8), which combines with its receptor in the endotheliocyte, facilitating further development of the inflammation and up-regulate the expression of adhesion molecules such as inter-cellular adhesion molecule 1 and lymphocyte function-associated antigen-1. IL-8 may interact with HP0638, the outer inflammatory protein, augmenting the adherence capacity of *H. pylori* and increasing the colonization density^25^.

Our result showed that the high-protein/cholesterol pattern, which characterized by high intake of animal offal, animal blood, animal liver, fish, seafood, and poultry, was associated with a lower prevalence of *H. pylori* infection even though the association was not strong. The top food items of high-protein/cholesterol pattern are animal offal, animal blood, and animal liver which especially rich in selenium and vitamin A. The best-known function of selenium is to protect membranes from oxidative damage^26^. Plasma selenium levels were similar between *H. pylori* (+) gastritis and healthy controls, but in the gastric tissue selenium levels were significantly higher in *H. pylori* (+) gastritis in a previous study^27^. And there was significantly decrease in mucosal selenium levels in patients after successful *H. pylori* eradication therapy which means selenium accumulates in gastric when it is needed^27^. Moreover, vitamin D has emerged as a central regulator of host defense against infections^28^. A study indicated that the vitamin D_3_ decomposition product 1 (VDP1) exerts an antibacterial action against *H. pylori* but not against other bacteria. Treatment with VDP1 induced a collapse of cell membrane structures of *H. pylori* and ultimately lysed the bacterial cells^29^. Animal foods, especially animal offal, animal liver, fish, and seafood, are rich in vitamin D^28^. Therefore, high intake of these kinds of food may provide adequate vitamin D and decrease the prevalence of *H. pylori* infection. However, dietary patterns have more characteristics and are not sufficient to explain the associations between *H. pylori* infection and dietary patterns based on single nutrient intake, meaning that further studies are needed in this field. Moreover, previous studies demonstrated that a high intake of animal foods was associated with alcoholic fatty liver disease^30^, metabolic syndrome^31^, and diabetes^32^, suggesting it would not be always beneficial for health. Further studies should clarify the specific intake of such foods to effectively decrease the prevalence of *H. pylori* infection while also avoiding diseases caused by over intake.

We did not, however, observe the same associations between food items and *H. pylori* infection in our major food group analyses. It is because that foods and nutrients may act independently or may interact with one another, meaning that further research is needed to clarify the interactions of foods and nutrients in the relationship between dietary patterns and *H. pylori* infection.

We also found a negative association between higher level of education and *H. pylori* infection. This finding was in line with previous studies which showed that a lower education level was associated with a higher prevalence of *H. pylori* infection in adults^33^, pregnant women^34^, and children^35^. A study in Uganda^34^ suggested that education may influence personal and household hygiene practices which were associated with *H. pylori* infection. Unlike previous studies^33^,^34^, there was no association between household income and *H. pylori* infection in present study. It may be caused by the reason that most of the participants in our study were high-income and middle-income earners. A previous study demonstrated that the difference of *H. pylori* infection was significant only between participants with high-income and those with low-income^35^. According to the exchange rate in Dec. 5, 2015, one dollar is approximately equal to 6.4 Yuan. The average household income was about 1000 USD per month in Tianjin while most of our participants have a household income more than 1000 USD.

A few limitations are notable. First, because of the cross-sectional design of our study, we cannot assess the causal relationships between dietary patterns and the risk of *H. pylori* infection. For example, maybe individuals with greater health-care seeking behavior are more likely to have their *H. pylori* diagnosed, and thus change their dietary patterns. Second, because of the nature of the self-reporting questionnaire, recall bias exists, and the food intake may not be exact. Third, *H. pylori* infection status was evaluated solely with an *H. pylori*-specific IgG antibody without other confirmed assessments such as a urease breath test or a rapid urease test. The diagnosis of *H. pylori* infection may be not exact. Moreover, participants in this study are inhabitants in Tianjin (one of the big and developed cities in China) and most of them have higher socioeconomic status (education level and household income) than general Chinese populations. The high level of socioeconomic status could be a reason for the low prevalence of *H. pylori* infection in the present study. Thus, the presence of *H. pylori* infection is not completely in accordance with current *H. pylori* infection rates in China which are reaching 40–60% in adults^36^. Further research is needed to clarify the results in the present study. However, nearly all occupations are covered in this study, and we also included retired individuals living in residential communities. Thus, the sample population used here is representative of the general adult population in Tianjin. Despite these limitations, this is the first study to reveal the relationships between dietary patterns and *H. pylori* infection.

## Materials and Methods

### Participants

This cross-sectional study was based on the Tianjin Chronic Low-grade Systemic Inflammation and Health (TCLSIH) Cohort Study, which is a large prospective dynamic cohort study focusing on the relationships between chronic low-grade systemic inflammation and the health status of a population living in Tianjin, China^37^,^38^. General participants were recruited, while having their annual health examinations but not curing any disease at the Tianjin Medical University General Hospital-Health Management Center, the largest and most comprehensive physical examination center in Tianjin.

During the research period, there were 12096 participants with no history of *H. pylori* eradication therapy and gastrointestinal symptoms completed a comprehensive health examination and a study questionnaire reporting their personal information, dietary intake, lifestyles and health condition. We excluded participants who did not complete data collection (n = 633). Additionally, we excluded participants who had a history of cardiovascular disease (n = 828) or cancer (n = 228) because we considered cardiovascular disease and cancer could affect lifestyles of participants seriously. The final cross-sectional study population comprised 10407 participants for analyses. This study was conducted according to the guidelines laid down in the Declaration of Helsinki and all procedures involving human subjects were approved by the medical ethics committee of Institutional Review Board of the Tianjin Medical University with the reference number of ‘TMUhMEC 201430’. Written informed consent was obtained from all subjects. The methods of this study were carried out in accordance with the approved guidelines.

### Identification of dietary patterns

Dietary intake in last month was assessed using a modified version of the food frequency questionnaire (FFQ) that included 100 food items (the initial version of the FFQ included 81 food items^30^) with specified serving sizes. The FFQ included 7 frequency categories ranging from ‘almost never eat’ to ‘twice or more per day’ for foods and 8 frequency categories ranging from ‘almost never drink’ to ‘four or more times per day’ for beverages. The mean daily intake of nutrients was calculated by using an ad hoc computer program developed to analyze the questionnaire. The Chinese food composition tables^39^ were used as the nutrient database. The reproducibility and validity of the questionnaire were assessed in a random sample of 150 participants and living in Tianjin by comparing the data from the questionnaire with the data from 2 dietary questionnaires collected approximately 3 months apart and 4-day weighed dietary records (WDRs). Spearman rank correlation coefficient for energy intake between 2 food frequency questionnaires administered 3 months apart was 0.68. Correlation coefficients for food items (fruits, vegetables, fish, meat, and beverages) between 2 food frequency questionnaires administered 3 months apart ranged from 0.62 to 0.79 Spearman’s rank correlation coefficient for energy intake by the WDRs and the FFQ was 0.49. Correlation coefficients for nutrients (vitamin C, vitamin E, polyunsaturated fats, saturated fats, carbohydrate and calcium) by the WDRs and the FFQ ranged from 0.35 to 0.54. We applied factor analysis in order to generate major dietary patterns and factor loadings on all 100 food items. Varimax rotation^40^ was applied for greater interpretability. After evaluation of eigenvalues (greater than 1.0) and the scree test, 3 factors were determined. Food items with a factor loading greater than |0.30| were the main contributors to dietary pattern and representative of the character of each pattern. Factors were named descriptively according to the food items showing high loading (absolute value) with respect to each dietary pattern as follows: ‘high-carbohydrate/sweet’ pattern, ‘balanced’ pattern and ‘high-protein/cholesterol’ pattern.

### Diagnosis of *H. pylori* infection

Blood samples of participants were used for serology, detecting anti-*H. pylori* antibodies (IgG) by enzyme-linked immunosorbent assay (ELISA). The *H. pylori* urease Immunogold Testing kit was from Beijing Tian Hong Sig biotechnology Co., Ltd. The sensitivity, specificity, and agreement were 98.91%, 98.29%, and 98.51%, respectively and similar to a former study used ELISA to detecting anti-*H. pylori* antibodies (IgG), which reported sensitivity and specificity as 95% and 82%^41^.

### Assessment of other variables

Previous studies demonstrated that *H. pylori* infection was associated with socio-demographic conditions, such as age, sex, education, household income, and number of household^34^,^35^. Therefore, age, sex, education, household income, and living alone status were adjusted. Health status could also change one’s dietary habit and thus affect the associations between *H. pylori* infection and diet. Thus physical activity, metabolic syndrome, type 2 diabetes, and family history of diseases were adjusted. Furthermore, we additionally adjusted for other relevant variables such as smoking status, drinking status, and salt intake.

Anthropometric parameters (height, weight and waist circumference) were recorded using a standard protocol. Blood samples for the analysis of fasting blood glucose (FBG) and lipids were collected in siliconized vacuum plastic tubes. Fasting blood glucose (FBG) was measured by the glucose oxidase method, serum triglycerides (TG) were measured by enzymatic methods, and high-density lipoprotein cholesterol (HDL) was measured by the chemical precipitation method using reagents from Roche Diagnostics on an automatic biochemistry analyzer (Roche Cobas 8000 modular analyzer, Mannheim, Germany). Waist circumference was measured at the umbilical level with participants standing and breathing normally. Metabolic syndrome was defined in accordance with the criteria of the American Heart Association scientific statements of 2009^42^. Diabetes was defined in the latest recommendations from American Diabetes Association^43^. BMI was calculated as weight/height^2^ (Kg/m^2^). A detailed personal and family history of physical illness and current medications were noted from ‘yes’ or ‘no’ responses to relevant questions in a self-reported questionnaire. Physical activity (PA) in the most recent week was assessed using the short form of the International Physical Activity Questionnaire (IPAQ)^44^. For socioeconomic characteristics, educational level was measured by asking the question ‘what is the highest degree you earned?’ and was divided into 2 categories: <College graduate or ≥College graduate. Household income was measured by asking the question ‘What’s your family’s income a month’ and was divided into 2 categories: <10000 Yuan or ≥10000 Yuan. The subjects were also classified as living alone or living with others.

### Statistical analysis

All analysis were performed using the Statistical Analysis System 9.3 edition for Windows (SAS Institute Inc., Cary, NC, USA), and a *P*-value of 0.05 was considered to be statistically significant. Descriptive data are presented as the geometric mean (95% CI) for age, BMI, physical activity, salt intake and energy intake as these variables are not subject to normal distribution, and as percentages for categorical variables.

In order to characteristics of participants according to *H. pylori* infection status, continuous variables were examined using analysis of variance and chi-square test for categorical variables. Quartiles were categorized across the scores of each dietary pattern based on the distribution of the scores for all the participants and used for further analysis. Relationship between quartilecategories of dietary pattern scores and *H. pylori* infection status were examined using logistic regression by three different models. Odds ratios (OR) and 95% Confidence interval (CI) were calculated. Model 1 was used to calculate the crude OR, and model 2 was adjusted for age, sex, and BMI. Model 3 additionally adjusted for age, sex, BMI, smoking status, drinking status, physical activity, total energy intake, metabolic syndrome, type 2 diabetes, household incomes, educational levels, living alone, salt intake, and family history of diseases (cardiovascular, hypertension, hyperlipidemia, and diabetes). The variables which are not subject to normal distribution have been log-transformed before been included in the model. A linear trend cross increasing quartiles was tested using the median value of each quartile as a continuous variable based on linear regression. Model 3 was adjusted for analyses of major individual food groups: animal foods, fruits, vegetables, sugared beverages and snacks, and refined grain and grain products.

### Transparency Declaration

The lead author affirms that this manuscript is an honest, accurate, and transparent account of the study being reported, that no important aspects of the study have been omitted and that any discrepancies from the study as planned have been explained. The reporting of this work is compliant with the “strengthening the reporting of observational studies in epidemiology” statement.

## Additional Information

**How to cite this article**: Xia, Y. *et al*. Dietary Patterns are Associated with *Helicobacter Pylori* Infection in Chinese Adults: A Cross-Sectional Study. *Sci. Rep*. **6**, 32334; doi: 10.1038/srep32334 (2016).

## Figures and Tables

**Table 1:** Participant characteristics by *H. pylori* infection status^a^.

Characteristics	*H. pylori* infection status		*P* value^b^
	No (n = 7429; 71.4%)	Yes (n = 2978; 28.6%)	
Sex (male %)	54.4	60.9	<0.0001
Age (y)	40.1 (39.8, 40.3)^c^	43.2 (42.8, 43.7)	<0.0001
BMI (kg/m2)	24.3 (24.2, 24.4)	24.7 (24.6, 24.9)	<0.0001
Physical activity (Mets × hours/week)	9.8 (9.5, 10.1)	10.5 (10.0, 11.1)	0.01
Energy intake (kcal/d)	2216.1 (2173.6, 2259.3)	2255.5 (2184.2, 2329.0)	0.36
Salt intake (gram/d)	10.9 (10.8, 11.0)	11.2 (11.1, 11.3)	<0.0001
Metabolic syndromes (%)	25.9	30.7	<0.001
Type 2 diabetes (%)	5.7	6.9	0.03
Smoking status (%)			
Smoker	14.3	15.1	0.28
Ex-smoker	4.9	5.5	0.16
Drinker (%)			
Everyday	4.1	5.4	<0.01
Sometime	59.6	60.7	0.30
Ex-drinker	7.8	7.7	0.90
Family history of diseases (%)			
CVD	30.6	31.8	0.22
Hypertension	47.3	48.7	0.20
Hyperlipidemia	0.4	0.4	0.60
Diabetes	21.2	22.9	0.05
Education (≥ College graduate, %)	67.1	61.8	<0.0001
Household income (≥10,000 Yuan, %)	32.0	31.4	0.55
Living alone (yes, %)	9.5	8.7	0.18

^a^*H. pylori, Helicobacter pylori*; BMI, body mass index; CVD, cardiovascular disease.

^b^Analysis of variance or chi-square test.

^c^Geometric mean (95% confidence interval) (all such values).

**Table 2:** The factor loadings scores^a^ of primary food groups of dietary patterns.

High-carbohydrate/sweet pattern		Balanced pattern		High-protein/cholesterol pattern	
Food and food groups	Factor loadings	Food and food groups	Factor loadings	Food and food groups	Factor loadings
pineapple	0.65	celery	0.65	animal offal (except for animal liver)	0.70
strawberry, kiwi fruit, persimmon	0.64	pumpkin, carrot	0.64	preserved egg	0.64
grape	0.61	cucumber	0.62	animal liver	0.64
peach	0.58	Chinese watermelon	0.61	animal blood	0.63
pear	0.57	Chinese cabbage	0.61	sausage	0.58
sweets, candied fruits	0.57	eggplant	0.59	instant noodle	0.56
watermelon	0.55	green vegetable	0.54	sea fish	0.56
Chinese cakes	0.54	radish (expect for carrot)	0.53	pork skin	0.55
banana	0.54	mushroom	0.52	freshwater fish	0.55
salted eggs	0.54	tomato (including the ketchup)	0.51	wonton	0.52
western-style pastry, cakes	0.54	sweet potato	0.49	seafood (shellfish, squid, shrimp)	0.52
ice cream	0.53	bell peppers	0.48	white wine	0.49
fruit juice, vegetable juice	0.52	raw vegetables (except for tomato)	0.48	carbonated beverage	0.45
preserved bean curd	0.52	potato (except for sweet potato)	0.48	miscellaneous sauce noodles	0.45
Chinese sauerkraut	0.52	coarse cereals	0.46	beer	0.45
red wine	0.50	lotus root	0.45	steamed stuffed bun, dumpling	0.41
cookies	0.50	hot pepper	0.43	red wine	0.40
white wine	0.49	onion	0.42	bread	0.40
sea-plant	0.49	other types of beans	0.42	sweets, candied fruits	0.40
other kinds of fruit	0.48	soya bean products	0.41	strong liquor	0.40

^a^For simplicity, only the top 20 food groups of factor loading scores of each pattern are shown.

**Table 3:** Adjusted relationships of quartiles of factor scores to *H. pylori* infection^a^.

Dietary patterns	Quartiles of factor scores (range, n = 10407)				*P* for trend^b^
High-carbohydrate/sweet pattern	Level 1 (−6.8, −0.4)	Level 2 (−0.4, −0.1)	Level 3 (−0.1, 0.2)	Level 4 (0.2, 15.1)	
No. of *H. pylori* infection	739	722	746	771	
Crude	Ref	0.94 (0.83, 1.01)^c^	1.02 (0.90, 1.15)	1.10 (0.97, 1.25)	0.06
Age-, sex and BMI-adjusted	Ref	0.97 (0.85, 1.10)	1.03 (0.91, 1.17)	1.11 (0.98, 1.27)	<0.01
Multiple adjusted^d^	Ref	1.26 (0.98, 1.62)	1.30 (1.01, 1.67)	1.65 (1.27, 2.17)	<0.001
Balanced pattern	Level 1 (−5.2, −0.6)	Level 2 (−0.6, −0.2)	Level 3 (−0.2, 0.4)	Level 4 (0.4, 9.4)	
No. of *H. pylori* infection	719	753	747	759	
Crude	Ref	1.08 (0.95, 1.23)	1.06 (0.93, 1.20)	1.08 (0.95, 1.22)	0.36
Age-, sex and BMI-adjusted	Ref	1.06 (0.94, 1.21)	1.02 (0.90, 1.16)	1.01 (0.89, 1.16)	0.53
Multiple adjusted^d^	Ref	0.89 (0.69, 1.14)	0.82 (0.63, 1.06)	0.92 (0.68, 1.23)	0.52
High-protein/cholesterol pattern	Level 1 (−4.6, −0.4)	Level 2 (−0.4, −0.1)	Level 3 (−0.1, 0.2)	Level 4 (0.2, 11.6)	
No. of *H. pylori* infection	788	730	756	704	
Crude	Ref	0.89 (0.78, 1.00)	0.95 (0.84, 1.07)	0.83 (0.73, 0.95)	0.01
Age-, sex and BMI-adjusted	Ref	0.93 (0.82, 1.06)	0.99 (0.88, 1.14)	0.88 (0.77, 1.00)	0.10
Multiple adjusted^d^	Ref	0.98 (0.76, 1.26)	0.95 (0.74, 1.23)	0.75 (0.57, 0.98)	0.04

^a^*H. pylori*, *Helicobacter pylori*.

^b^Multiple logistic regression analysis.

^c^Odds ratios (95% confidence interval) (all such values).

^d^Adjusted for age, sex, BMI, smoking status, drinking status, physical activity, total energy intake, metabolic syndrome, type 2 diabetes, household incomes, educational levels, living alone, salt intake, and family history of diseases (cardiovascular, hypertension, hyperlipidemia, and diabetes).

**Table 4:** Adjusted relationships of quartiles of individual food group intake to *H. pylori* infection^a^.

Food groups	Quartiles of individual food group intake (gram/day, n = 10407)					*P* for trend^b^
animal foods	No. of *H. pylori* infection	741	776	738	723	0.53
	Range	Level 1 (0, 149)^c^	Level 2 (149, 205)	Level 3 (205, 280)	Level 4 (280, 2092)	
	Multiple adjusted^d^	ref	1.04 (0.81, 1.34)	0.98 (0.76, 1.28)	0.93 (0.69, 1.25)	
fruits	No. of *H. pylori* infection	771	721	727	759	
	Range	Level 1 (0, 99)	Level 2 (99, 162)	Level 3 (162, 255)	Level 4 (255, 1743)	0.09
	Multiple adjusted^d^	ref	1.06 (0.82, 1.35)	1.00 (0.77, 1.30)	1.30 (0.98, 1.74)	
vegetables	No. of *H. pylori* infection	736	733	762	747	
	Range	Level 1 (0, 280)	Level 2 (280, 392)	Level 3 (392, 560)	Level 4 (560, 3138)	0.68
	Multiple adjusted^d^	ref	1.02 (0.79, 1.31)	0.93 (0.71, 1.21)	0.96 (0.71, 1.31)	
sugared beverages and snacks	No. of *H. pylori* infection	830	736	681	731	
	Range	Level 1 (0, 12)	Level 2 (12, 43)	Level 3 (43, 87)	Level 4 (87, 1569)	0.14
	Multiple adjusted^d^	ref	0.94 (0.74, 1.21)	0.98 (0.76, 1.27)	1.18 (0.90, 1.55)	
refined grain and grain products	No. of *H. pylori* infection	742	747	764	725	
	Range	Level 1 (0, 168)	Level 2 (168, 230)	Level 3 (230, 305)	Level 4 (305, 1394)	0.15
	Multiple adjusted^d^	ref	0.90 (0.69, 1.17)	0.93 (0.72, 1.21)	0.79 (0.59, 1.05)	

^a^*H. pylori, Helicobacter pylori*.

^b^Multiple logistic regression analysis.

^c^Adjusted odds ratios (95% confidence interval) (all such values).

^d^Adjusted for age, sex, BMI, smoking status, drinking status, physical activity, total energy intake, metabolic syndrome, type 2 diabetes, household incomes, educational levels, living alone, salt intake, and family history of diseases (cardiovascular, hypertension, hyperlipidemia, and diabetes).
